# The association between social isolation and musculoskeletal health in older community-dwelling adults: findings from the Hertfordshire Cohort Study

**DOI:** 10.1007/s11136-021-02784-7

**Published:** 2021-02-17

**Authors:** Gregorio Bevilacqua, Karen A. Jameson, Jean Zhang, Ilse Bloom, Kate A. Ward, Cyrus Cooper, Elaine M. Dennison

**Affiliations:** 1 MRC Lifecourse Epidemiology Unit, University of Southampton, Southampton General Hospital, Southampton, UK; 2 MRC Nutrition and Bone Health Group, Cambridge, UK; 3 National Institute for Health Research Musculoskeletal Biomedical Research Unit, University of Oxford, Oxford, UK; 4 Victoria University of Wellington, Wellington, New Zealand

**Keywords:** Social isolation, BMD, Physical capability, Depression, Anxiety, Older adults

## Abstract

**Purpose:**

Social isolation has been associated with both physical and psychological adverse outcomes and is prevalent in older adults. We investigated the impact of social isolation on bone mineral density (BMD) and physical capability in community-dwelling older adults.

**Methods:**

Data were collected in 2011 and 2017 from the Hertfordshire Cohort Study. In 2011, we assessed social isolation using the six-item Lubben Social Network Scale (LSNS-6) and the Maastricht Social Participation Profile (MSSP) and depressive and anxiety symptoms using the Hospital Anxiety and Depression Scale (HADS). Physical capability was assessed by performing tests of gait speed, chair stands, timed up and go and balance at both time points. BMD was assessed using dual X-ray absorptiometry (DXA) at both time points.

**Results:**

Data were available from 369 participants in 2011 and 184 in 2017. Forty percent of men and 42.4% of women were socially isolated. Isolated participants had higher odds of depressive disorder (OR 3.01, 95% CI 1.27–7.11, *p* < 0.02). Social isolation at baseline was associated with poor physical capability scores at follow-up (OR 5.53, 95% CI 1.09–27.99, *p* < 0.04). No associations were found between social isolation and BMD at either time point.

**Conclusions:**

Social isolation was associated with higher odds of having depressive symptoms and predicted the development of poor physical capability 6 years later. Further longitudinal studies that include loneliness as a covariate are warranted.

## Background

Social relationships play a fundamental role in individuals’ lives and health and have previously been associated with physical and psychological wellbeing [1]. Social isolation is defined as the scarceness or absence of regular social contacts and relationships with relatives, friends and neighbours and lack of social connection and involvement with the wider society; such infrequency of contact with one’s social network can be objectively measured [2–4]. Social isolation is thus different from loneliness, which is a subjective, negative evaluation of the discrepancy between an individual’s desired and actual quantity and quality of social relationships [5–7]. Measures of social isolation typically include evaluations of the size of one’s social network, number of interactions with family members, friends and neighbours and level of participation in social organisations [8].

Risk factors for social isolation are numerous and include being 75 years and older, living alone, having limited financial resources, having poor mental and/or physical health, being part of a minority group and having no children [9]. While social isolation can lead to loneliness in some people, individuals can feel lonely without being socially isolated or conversely be isolated without feeling lonely [5]. Social isolation has been associated with higher risks of a number of both physical and psychological adverse health outcomes, including myocardial infarction, stroke, depression and even increased mortality [10–14].

Previous studies have found that social isolation is prevalent in the elderly and that the number of older adults at risk of becoming isolated is currently increasing [15, 16]. Given the burden of musculoskeletal disease in older adults, especially sarcopenia and osteoporosis [17] it is notable that very few studies have considered whether social isolation is also associated with poor musculoskeletal health in older adults. We were interested to study this issue because in addition to the adverse health outcomes cited above, in older adults social isolation has also been associated with lifestyle factors linked to poorer musculoskeletal health, including poor nutrition, increased sedentary behaviour and less frequent physical activity [3], which may be as a consequence of, or co-exist with, depressive mood, itself common in socially isolated individuals. While a recent study addressed potential associations of being socially isolated with frailty and depression, it did so by measuring physical capability exclusively by the single gait speed test [18].

Hence in the present article, we investigated whether objectively measured social isolation in a cohort of well phenotyped community-dwelling older adults in the UK was cross-sectionally associated with lifestyle, bone mineral density (BMD) or physical capability, considering whether any associations observed could be ascribed to increased depressive and anxiety symptoms in isolated individuals. Furthermore, we performed a longitudinal analysis to consider whether social isolation led to worse bone health, or altered physical capability, a mean of 6 years later.

## Methods

**Fig. 1:**
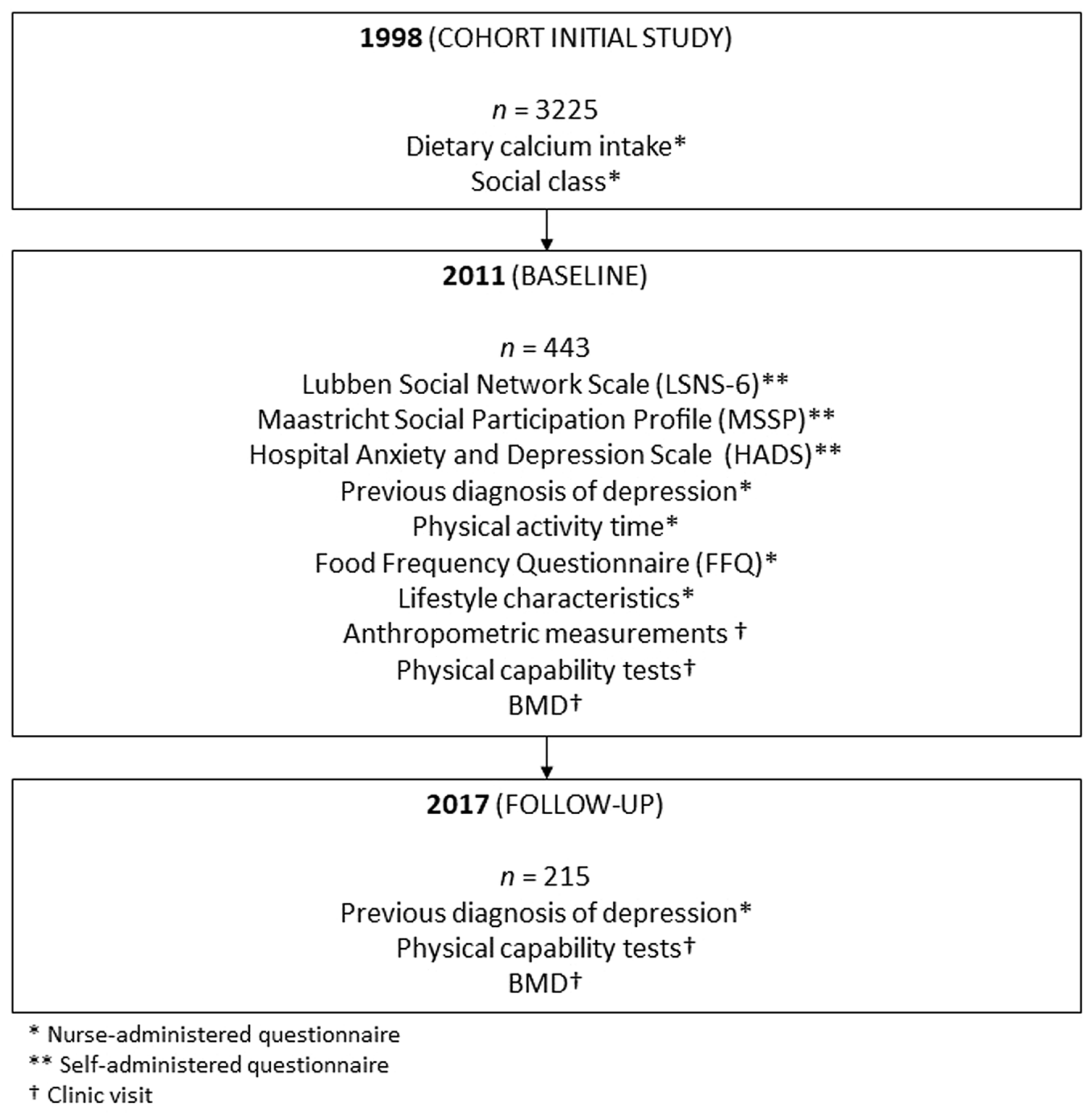
Study flowchart and timeline

The Hertfordshire Cohort Study (HCS) is a population-based sample of men and women born between 1931–9 in Hertfordshire and originally recruited in order to study the relationship between growth in infancy and the subsequent risk of adult diseases [19, 20]. Participants have been seen at several time points; Fig. 1 shows the information collected at each time point and the number of participants.

The Hertfordshire Cohort Study was established in 1998 [19, 20]. In 2011, having provided written informed consent, 443 HCS participants (222 men and 221 women) who had formed part of the initial cohort and who were selected based on geographic location (East Hertfordshire) only were visited at home by a trained fieldworker, when a questionnaire was completed. The visits also included measurements of height and weight to calculate body mass index (BMI) and the performance of a number of physical capability tests. A previous diagnosis of depression was self-reported, both at baseline and follow-up, and assessed asking the question: ‘Have you been told by a doctor that you have any of the following conditions?’, with depression being one of the conditions recorded. Participants were also invited to attend a research clinic where BMD was assessed using a dual X-ray absorptiometry (DXA) scan. In 2017, 215 participants (112 men and 103 women) of this original group were invited to participate in a follow-up study and re-attended for a further DXA scan and physical capability tests.

### Exposure: social isolation

Social isolation was assessed using the six-item Lubben Social Network Scale (LSNS-6) [21] and the Maastricht Social Participation Profile (MSPP) [22]. The LSNS-6 tool measures the number and frequency of social interactions with friends (three items) and family members (three items). Each answer is assigned a score ranging from 0 (“none”) to five (“nine or more”), and the overall final score ranges from 0 (indicating high isolation or few social resources) to 30 (indicating low isolation or many social resources). The LSNS-6 was chosen as it has been shown to have good internal consistency across samples of community-dwelling older adults [21, 23, 24].

The MSPP measures an individual’s actual social participation over a period of four weeks [22], and consists of three indexes: consumptive participation (which refers to organised activities and includes six items); formal social participation (which refers, for example, to volunteer activities and includes three items); and informal social participation (which refers to contacts with family members, friends and acquaintances). Answers are classified using a Likert-type scale from zero (“not at all”) to three (“more than twice a week”). Two types of scores are then derived for each index: diversity (the number of items on which a respondent scored at least one) and frequency (mean score of the items). Higher scores are considered indicative of more diverse or more frequent social participation. The MSPP was chosen as it has been shown to have good validity and reproducibility [22, 25].

Since the informal social participation index of the MSPP is similar to the LSNS-6, we used the latter, together with its cut-off value [26, 27]. A total diversity score was calculated considering the consumptive participation and the formal social participation indexes of the MSPP. A participant’s diversified social participation index was calculated considering the median value of each of the following: total diversity, consumptive participation diversity and frequency and formal social participation diversity and frequency.

Social isolation was defined as a LSNS-6 score < 12, in accordance with Lubben and colleagues, [21], or ≤ the median of all five of the MSPP scores [27]. Participants were identified as socially isolated on either one or the other scale.

### Outcomes

BMD was assessed using a DXA scan (baseline, 2011: Lunar Prodigy Advanced Scanner, GE Medical Systems, UK; follow-up, 2017: GE Lunar iDXA, GE Healthcare, UK). Bilateral scans of the proximal femur were taken, with areal BMD (g/cm^2^) of the non-dominant hip used for analysis; if participants had previously fractured or had hip arthroplasty, the contralateral hip was used. All scans were acquired by a trained technician using standard positioning techniques and in accordance with the manufacturer’s instructions [28, 29].

Physical capability was assessed at both time points using the following Short Physical Performance Battery (SPPB) tests: gait speed, timed up and go, chair rises and tandem stands [30]. We also measured handgrip strength, which was assessed three times for each hand using a Jamar dynamometer; the maximum measurement was used for analysis [31]. We chose to use the SPPB as SPPB scores are strongly correlated with measures of physical fitness in older adults [32], and the test–retest reliability of the SPPB has been demonstrated to be high in many studies [33, 34]. Furthermore, a systematic review of 12 functional assessment instruments for older adults identified the SPPB as the most positive overall rating and the highest scores on reliability (intra-class correlation coefficients ranging from 0.70 to 0.99), validity and responsiveness to change [35].

We measured gait speed using an eight-foot course with no obstructions for an additional foot at either end. Participants were asked to walk at their customary pace and the time taken was recorded using a stopwatch. The use of assistive devices, such as canes, was permitted if necessary. We determined gait speed by dividing the distance traversed by the time between the first and last step.

In the timed up-and-go test, participants were asked to rise from a chair as quickly as possible, walk three metres at a comfortable and safe pace, turn around, walk back to the chair and sit down again. The use of mobility aids was permitted if required according to usual practice for these tests [36], but 93% of participants did not use any. Time taken was recorded using a stopwatch.

To test chair rises, participants crossed their arms across their chest and stood up. Those who could complete this task were asked to stand up and sit down again a total of five times. The time was taken from their initial sitting position until they were standing on the fifth repetition.

The tandem stands tested the participants’ ability to maintain their balance. The standing balance test involved a semi-tandem stand where participants placed one foot in front of the other such that the big toe of one foot was touching the side of the heel of the other. If participants could not hold the semi-tandem stand for 10 s, they did a side-by-side stand (standing with the feet side-by-side). If they could hold the semi-tandem stand for 10 s, they also attempted a full tandem stand where participants placed one foot in front of the other (touching heel to toe) and held this position for as long as they could up to 10 s.

A physical capability score was derived from the tests of gait speed, chair rises and balance, according to the SPPB scoring guidelines [30]. For the walking test and the chair rise test, those participants who could not complete the test were given a score of 0. The remaining participants’ times were divided into quartiles and given a score of 1–4, slowest to fastest quartile. For the balance test, if participants could maintain balance in the full tandem stand for a minimum of 10 s, they were given a score of 4; if their time was ≥ 3 and < 10 s, they scored 3; if they maintained balance for less than 3 s but were able to maintain a semi-tandem stand, they scored 2; if they could not do the semi-tandem stand but could do the side-by-side stand, they scored 1; and if they could do neither the semi-tandem nor the side-by-side stand, they scored 0.The scores for the walking test, chair rises and balance test were then summed. The maximum possible score was 12 and the minimum was 0. Scores lower than 9 were considered to be indicative of poor physical capability.

Depressive and anxiety symptoms reported at baseline were assessed via questionnaire and measured using the Hospital Anxiety and Depression Scale (HADS) [37]. A possible case of any depressive or anxiety disorder was defined as a HAD-D (depression) or HAD-A (anxiety) score, respectively, between 8 and 10 and a probable case as a score ≥ 11 [37]. The HADS is a self-assessment scale that has been found to perform well in assessing symptom severity, and case assessment of anxiety disorders and depression in both somatic, psychiatric and primary care patients, as well as in the general population [38]. The HADS is a validated tool, with good correlations between the two subscales (mean 0.56) and good internal consistency (Cronbach's alpha for HADS-A: mean 0.83; and for HADS-D: mean 0.82); sensitivity and specificity for both HADS-A and HADS-D of approximately 0.80 were very similar to the sensitivity and specificity achieved by the General Health Questionnaire, and correlations between HADS and other commonly used questionnaires ranged between 0.49 and 0.83 [38].

### Covariates

Covariates were assessed via nurse- and self-administered questionnaire at each time point (see Fig. 1). Physical activity time was self-reported (using the Dallosso questionnaire) and calculated as the average minutes per day spent walking, cycling, gardening, playing sport and doing house work in the last two weeks [39]. We also collected information on lifestyle parameters (i.e. smoker status and alcohol consumption). Diet was assessed using an administered food frequency questionnaire (FFQ). A ‘prudent’ diet score was calculated for each participant based on their consumption of 24 indicator foods and was used as a measure of diet quality Higher prudent diet scores indicate healthy diets, characterised by higher consumption of fruit, vegetables, whole grain cereals and oily fish and low consumption of white bread, added sugar, full-fat dairy products, chips and processed meat [40, 41]. Dietary calcium intake was obtained from the HCS questionnaire administered in the first pass of the study, as well as social class, which was determined from the participants’ current or most recent occupation for men and never-married women, and of the husband for married women; occupations were was classified as non-manual (classes I-IIINM) or manual (classes (IIIM-V) according to the 1990 OPCS Standard Occupational Classification scheme.

## Statistical analysis

Descriptive statistics for continuous variables were expressed as mean and standard deviation (SD) or median and interquartile range (IQR) as appropriate. Categorical variables were expressed as frequency (N) and percentage (%). Characteristics were presented for men and women separately and differences between the sexes were assessed using Student’s t-tests, Mann–Whitney U tests, Pearson’s χ^2^ tests or Fisher’s exact tests, as appropriate. The BMD and physical capability continuous outcomes were transformed to Fisher-Yates (FY) z-scores using the Fisher-Yates rank-based inverse normal transformation to normalise the data. Linear, logistic and ordered logistic regression analyses were used to examine the associations between social isolation and physical capability and depression and anxiety symptoms. Since no significant sex interactions were found, the regression analyses were conducted with men and women pooled and adjusted for sex. The regression analyses were undertaken with and without adjusting for the following confounders: age, BMI, smoker status (never, ex-smoker or current smoker), alcohol consumption (units per week), physical activity time, prudent diet score, and additionally, for the physical capability and BMD analyses, dietary calcium and follow-up time (for longitudinal analyses). Our selection of confounders was based on pre-existing knowledge of factors known to impact bone and muscle health. Analyses were performed with and without adjustment for social class. Participants were excluded from those analyses for which they did not have the required data, but they continued to be included in those analyses for which they did have the required data. The analyses were conducted using Stata, version 16. A *p*-value of ≤ 0.05 was considered to be statistically significant.

## Results

Data on social isolation were available for 369 participants (185 men and 184 women) at baseline; of these, 184 participants (94 men and 90 women) also attended at follow-up 6 years later. Table 1 provides the demographic characteristics of the participants included in the baseline study. Participants who provided baseline and follow-up data were slightly younger, more active, had higher grip strength and performed better on physical capability tests; they also had slightly lower baseline dietary calcium intake and drunk more alcohol compared to those who completed the baseline study only (data not shown). The mean (SD) age of participants in 2011 was 75.5 (2.5) years for men and 75.7 (2.6) years for women. Women tended to have higher prudent diet scores (mean (SD): 0.43 (1.43) for women, -0.14 (1.59) for men) and had dedicated more time to physical activity over the previous two weeks than men (median (IQR): 201 min/day (137–287) for women, 181 min/day (105–266) for men); they also had lower daily calcium intake (median (IQR): 1092 mg (934–1270) for women, 1236 mg (1022–1425) for men), consumed less alcohol per week (median (IQR): 0.5 units (0.0–4.0) for women, 6.9 units (1.0–14.0) for men), and were less prone to be, or have been, a smoker than men, with 63.6% of them reporting to have never smoked compared to only 40.5% of men who said they have never smoked. Lastly, women had slightly higher scores on the HAD-A scale (median (IQR): 5 (3–7) for women, and 4 (2–6) for men) and more possible (15.2% of women and 9.2% of men) and probable cases (7.6% of women and 3.8% of men) of anxiety than men. All these sex differences were statistically significant. Men had higher grip strength, gait speed and physical capability overall scores than women, with 59.1% of male participants returning a score ≤ 9 (indicative of poor physical capability) compared to 69.3% of women who scored below this threshold. Similarly, 26.1% of women could not maintain balance for at least 10 s during the tandem stand test, compared to only 16.8% of men who could not complete the test (all *p* ≤ 0.05).

**Table 1:** Baseline characteristics of study participants, physical capability, social isolation, depression and HAD scores and cases

	Men			Women				
	*N*	Mean	SD		*N*	Mean	SD	*p*-value
Age (yrs)	185	75.5	2.5		184	75.7	2.6	0.388
Prudent diet score	184	− 0.14	1.59		184	0.43	1.43	< 0.001
Height (cm)	184	173.0	6.5		181	158.9	6.0	< 0.001

		Median	IQR			Median	IQR	
Weight (kg)	184	81.8	74.8–89.5		184	70.8	63.8–79.7	< 0.001
BMI (kg/m^2^)	184	27.5	25.3–29.8		181	28.6	25.1–31.7	0.079
Activity time in last 2 weeks (min/day)	172	181	105–266		175	201	137–287	0.045
Dietary calcium intake (mg/day)^a^	185	1236	1022–1425		184	1092	934–1270	< 0.001
Alcohol consumption (units/week)	185	6.9	1.0–14.0		184	0.5	0.0–4.0	< 0.001

	Total *N*	*N*	%		Total *N*	*N*	%	
Smoker status	185				184			< 0.001
Never		75	40.5			117	63.6	
Ex		102	55.1			63	34.2	
Current		8	4.3			4	2.2	
Social class^a^	176				184			0.637
I-IIINM		76	43.2			84	45.7	
IIIM-V		100	56.8			100	54.3	
Social isolation	185	74	40		184	78	42.4	0.641

Physical capability								
	*N*	Mean	SD		*N*	Mean	SD	
Maximum grip (kg)	184	36.4	7.3		184	21.6	6.0	< 0.001
Gait speed (m/s)	173	0.79	0.17		174	0.74	0.19	0.006

	Total *N*	*N*	%		Total *N*	*N*	%	
Tandem stand < 10 s	179	30	16.8		176	46	26.1	0.031

	*N*	Median	IQR		*N*	Median	IQR	
6 m Timed up and go (sec)	172	11.4	10.1–13.0		173	11.8	10.0–14.0	0.304
Chair rise time (secs)	164	15.8	13.9–18.9		155	16.6	13.6–19.6	0.396
Physical capability score	171	9.0	7.0–11.0		166	8.0	6.0–10.0	0.016

	Total *N*	*N*	%		Total N	*N*	%	
Low phisycal capability score (< = 9)	171	101	59.1		166	115	69.3	0.051

Depression and anxiety								
	*N*	Median	IQR		*N*	Median	IQR	
Had-d score	184	3	1–5		184	3	1–5	0.605
Had-a score	184	4	2–6		184	5	3–7	0.008

	Total *N*	*N*	%		Total *N*	*N*	%	
Had-d category	184				184			0.200
0–7 Non-case		174	94.6			165	89.7	
8–10 Possible case		6	3.3			13	7.1	
11 + Probable case		4	2.2			6	3.3	
Had-a category	184				184			0.047
0–7 Non-case		160	87.0			142	77.2	
8–10 Possible case		17	9.2			28	15.2	
11 + Probable case		7	3.8			14	7.6	
Self-reported depression	185	13	7.0		184	18	9.8	0.340

^a^ Data obtained from the first pass of the HCS study (1998)

Social isolation was high in our sample of community-dwelling adults: 40.0% of men and 42.4% of women were identified as socially isolated on either the LSNS-6 or MSPP scales. Seven percent of men and 9.8% of women reported a diagnosis of depression by a doctor. These sex differences were not statistically significant. Table 2 shows lifestyle characteristics by social isolation; isolated subjects recorded a lower prudent diet score (mean (SD): -0.12 (1.45)) than those who were not socially isolated (mean (SD): 0.33 (1.57)), but there was no significant difference in mean daily dietary calcium intake.

**Table 2:** Baseline participants’ characteristics by social isolation

	Non-isolated			Isolated*			
	*N*	Mean	SD	*N*	Mean	SD	*p*-value
Age (yrs)	217	75.5	2.6	152	75.8	2.5	0.289
Prudent diet score	216	0.33	1.57	152	− 0.12	1.45	0.005
Height (cm)	215	166.2	9.0	150	165.7	10.2	0.631

	*N*	Median	IQR	*N*	Median	IQR	*p*-value
Weight (kg)	217	77.1	69.1–87.5	151	76.3	67.7–85.4	0.543
BMI (kg/m^2^)	215	28.1	25.6–30.6	150	27.3	24.5–31.0	0.549
Activity time in last 2 weeks (min/day)	206	189	132–268	141	197	111–286	0.778
Daily dietary calcium intake (mg)^a^	217	1170	1003–1350	152	1147	925–1361	0.324
Alcohol consumption (units/week)	217	3.1	0.3–9.0	152	1.4	0.1–7.7	0.094

	Total *N*	*N*	%	Total *N*	*N*	%	*p*-value
Sex	217			152			0.641
Male		111	51.2		74	48.7	
Female		106	48.8		78	51.3	
Smoker status	217			152			0.980
Never		112	51.6		80	52.6	
Ex		98	45.2		67	44.1	
Current		7	3.2		5	3.3	
Social class^1^	212			148			0.962
I-IIINM		94	44.3		66	44.6	
IIIM-V		118	55.7		82	55.4	

^*^Social isolation defined as LSNS-6 score < 12 or ≤ the median of the MSPP scores

^a^Data obtained from the first pass of the HCS study (1998)

Isolated participants had a higher odds of depressive disorder (HAD-D) when results were adjusted for sex only (OR 2.93, 95% CI 1.32–6.51, *p* = 0.008). The association remained significant after adjustment for sex, age, BMI, smoker status, alcohol consumption, physical activity time and prudent diet score (OR 3.01, 95% CI 1.27–7.11, *p* < 0.02). When we further adjusted for social class, the association persisted. However, low numbers of incident cases of depression prevented us being able to assess whether social isolation led to subsequent depression.

In initial cross-sectional analyses, we found no significant relationships between social isolation and physical capability. However, after excluding those participants who recorded a low physical capability score at baseline, baseline social isolation was associated with development of a poor physical capability score after 6 years (OR 3.49, 95% CI 1.14–10.68, *p* < 0.03), with analyses robust to adjustment (OR 5.53, 95% CI 1.09–27.99, *p* < 0.04) when social class was included in the adjustments (see Table 3). By contrast, we found no association between BMD and social isolation at either time point, before or after adjustments (including adjusting for taking osteoporosis medication) at baseline.

**Table 3:** Social isolation as explanatory variable for physical capability

all participants in 2017								
	Adjusted for sex and follow-up time only				Fully adjusted^a^			
	*N*	Regression coefficient	95% CI	*p*-value	*N*	Regression coefficient	95% CI	*p*-value
Grip strength (fy z-score)	183	0.13	(− 0.16, 0.42)	0.362	166	0.12	(− 0.19, 0.44)	0.445
Gait speed (fy z-score)	183	-0.12	(− 0.41, 0.16)	0.397	166	− 0.05	(− 0.37, 0.26)	0.736
6 m Timed up and go (fy z-score)	181	0.06	(− 0.23, 0.35)	0.698	165	0.01	(− 0.30, 0.32)	0.970
Chair rise time (fy z-score)	156	0.13	(− 0.17, 0.44)	0.396	140	0.02	(− 0.32, 0.36)	0.915
Physical capability score (fy z-score)	171	-0.05	(− 0.38, 0.28)	0.760	156	0.03	(− 0.33, 0.39)	0.853

	*N*	Odds ratio	95% CI	*p*-value	*N*	Odds ratio	95% CI	*p*-value
Low physical capability score (≤ 9)	171	2.02	(1.00, 4.09)	0.051	151	1.64	(0.72, 3.77)	0.241
Tandem stand < 10 s	151	1.34	(0.61, 2.95)	0.461	136	1.01	(0.40, 2.50)	0.991

Excluding those with low physical capability in 2011								
	*N*	Regression coefficient	95% CI	*p*-value	*N*	Regression coefficient	95% CI	*p*-value
Grip strength (fy z-score)	69	− 0.19	(− 0.69, 0.31)	0.450	63	− 0.04	(− 0.62, 0.53)	0.883
Gait speed (fy z-score)	69	− 0.29	(− 0.69, 0.11)	0.153	63	− 0.16	(− 0.61, 0.30)	0.490
6 m Timed up and go (fy z-score)	67	0.18	(− 0.26, 0.61)	0.413	62	0.08	(− 0.40, 0.56)	0.741
Chair rise time (fy z-score)	66	− 0.14	(− 0.55, 0.27)	0.504	60	− 0.08	(− 0.55, 0.39)	0.728
Physical capability score (fy z-score)	66	− 0.22	(− 0.68, 0.23)	0.334	61	− 0.26	(− 0.76, 0.25)	0.315

	*N*	Odds ratio	95% CI	*p*-value	*N*	Odds ratio	95% CI	*p*-value
Low physical capability score (≤ 9)	66	3.49	(1.14, 10.68)	0.029	59	5.53	(1.09, 27.99)	0.039
Tandem stand < 10 s	63	5.99	(1.08, 33.31)	0.041	57	18.14	(0.71, 462.73)	0.079

^a^Adjusted for sex, follow-up time, age, BMI, smoker status, alcohol consumption, physical activity time, prudent diet score and social class

## Discussion

We have found a high prevalence of social isolation in a population of older community-dwelling adults, and this was consistent with previous literature suggesting that 50% of the worldwide population aged over 60 is at risk of becoming socially isolated [42]. We also observed that baseline social isolation in 2011 predicted the development of poorer physical capability at follow-up 6 years later. By contrast we did not find longitudinal associations between social isolation and BMD in this study. No previous study, to our knowledge, has addressed potential effects of being isolated on musculoskeletal health. As expected, we found that being socially isolated was associated with depressive symptoms, but unfortunately low numbers of participants who reported new depression over the follow-up period prevented any investigation of temporal trend, i.e. whether social isolation led to depression.

The fact that in our study BMD was not related to social isolation may reflect the crude tool we used to assess social isolation; in our cohort, there was no significant difference in time dedicated to physical activity, nor in dietary calcium intake, between isolated and non-isolated participants, despite social isolation being an established barrier to physical activity in some other studies [43]. Our observation that social isolation in this study was associated with poorer diet quality but no difference in daily calcium intake (see Table 2), which may be considered a stronger determinant of bone health, may help to explain the relationships we observed. Although weight bearing physical activity might be expected to impact BMD, as reported above, we did not identify differences in physical activity levels between isolated and non-isolated participants. This observation is in contrast to some other reported data. In the English Longitudinal Study of Ageing (ELSA), a UK cohort study of more than 3000 men and women significantly younger than our own (i.e. aged ≥ 52 years), isolated men and women tend to be less physically active, and to consume less than 5 servings per day of fruits and vegetables [40]. In another study using the same cohort, Schrempft and colleagues reported that social isolation was again linked to reduced daily physical activity as assessed by wrist worn accelerometer [3].

In this study we found that being socially isolated was associated with higher odds of having a low physical capability score at follow-up 6 years later. Reduced physical capability in later life is associated with a number of adverse outcomes, such as increased propensity to fall and inability to self-care [44, 45], nursing home and hospital admissions [46, 47]. Our results were robust to adjustment for both diet and physical activity, raising the possibility of residual confounding, and further research is now required to study these relationships further. Consistent with our own observations, a population sample of 1020 Lebanese older men and women (mean (SD) age 74.9 (6.9)) [11] previously reported that social isolation was associated with malnutrition. A possible reason as to why being socially isolated may impact diet quality in older women in our UK cohort setting in our study is that with widowhood or in general the absence of someone to cook for, some women may have changed their dietary habits [48–52]. Our findings are consistent with those of Bloom et al., who found associations between various social factors and better diet quality in a subgroup of the HCS: in particular, better diet quality was associated with larger social networks in women but not in men, while no associations between poor diet quality and depression or anxiety were found in women [53].

We acknowledge that our assessment of social isolation in this study is crude; preferable would been a qualitative element with more detailed responses regarding the subjective experience. As described earlier, some people may objectively be socially isolated but not feel lonely, while other may feel lonely despite being less socially isolated. It is possible that loneliness [4, 7] is more important than social isolation in the age group considered in our study [54]. Loneliness is indeed associated with poor physical and mental health outcomes [55–57], reduced quality of life [58, 59], as well as raised blood pressure and physical inactivity [60]. It has been previously demonstrated that social isolation and loneliness are weakly correlated [61] and that loneliness can occur in the presence or absence of social isolation [62, 63]. Loneliness was not measured in this cohort, but will be an important component of future studies. Whereas both loneliness and social isolation can be caused by a multitude of factors, it is likely that social and emotional support from others may provide a protective effect, and interventions should, thus, aim at improving both the quality of social relationships and involvement in social activities [4].

Isolated participants in our study had cross-sectional higher odds of depressive disorder. Our results are consistent with previous studies that found associations between social isolation and depressive mood and syndrome [11], depressive symptoms [31] and depression [41]. Unfortunately, small numbers of participants reporting depressive symptoms prevented us from considering whether depression led to social isolation or vice versa, which would be important to consider in future work. Merchant and colleagues found that social isolation was associated with slower gait speed but not depression in a population sample of Asian community-dwelling adults aged 60 years and above [12]. Although these conflicting findings from otherwise very similar studies might be ascribed to cultural and lifestyle differences between Eastern and Western populations, it is possible that differences in the study design may also contribute to the different results of our investigation. Whereas both studies have assessed social isolation via the LSNS-6, we also assessed isolation via the MSPP. Moreover, Merchant and colleagues measured gait speed only, while our study used a validated train of tests to assess physical capability [30].

Our study has a number of limitations. The study population may not be entirely representative of the wider UK population, since all recruited participants were born in the county of Hertfordshire, were still living in their homes in their eighth decade, and were all Caucasian. However, we have previously demonstrated that this cohort is representative of the general population with regard to anthropometric body build and lifestyle factors, such as smoking, alcohol intake and dietary calcium intake, which was in line with data found in the European Investigation into Cancer and Nutrition Cohort (EPIC) [64]; as a result, selection bias was minimal [19]. We do acknowledge a ‘healthy’ responder bias among participants in serial waves within the HCS [19], with participants who completed the study being slightly younger and healthier than those who did not take part in both stages of this research. Social class was determined from the participants’ current or most recent occupation for men and never-married women, and of the husband for married women: this is a crude assessment which might not be reflective of participants’ actual occupation and, therefore, social class; however, having performed analysis with and without adjustment for social class, our results were not affected. An additional limitation of this study is its cross-sectional design of most of its analysis. Future studies may benefit from exploring whether social isolation is associated with longitudinal changes in physical capability and BMD. Our study has also a number of strengths. The LNS-6 provides a reliable measurement of social isolation; Rasch analysis showed unidimensionality of the overall scale, high person and item reliability and good fit of individual items with only one exception [65]. Similarly, it has been previously shown that the MSPP has good validity and acceptable reproducibility [22]. Moreover, the HAD Scale has been found to perform well in assessing symptom severity, and case assessment of anxiety disorders and depression in both hospitalised patients and the general population [38]. A significant strength of this study is the reasonably large sample size in a population of community-dwelling older adults that have been extensively phenotyped and well characterised with regard to lifestyle and past medical history.

## Conclusions

In a cohort of community-dwelling older adults in the UK, we found that social isolation as assessed by simple screening questions was associated with depression, and poorer diet, and also predicted the development of poor physical capability 6 years later. These findings suggest that simple screening questions may be used to identify older individuals who might benefit from targeted support to improve diet and reduce risk of adverse health outcomes subsequently.

## Data Availability

The datasets used and/or analysed during the current study are available from the corresponding author on reasonable request.
